# Atlas of the HIV-1 Reservoir in Peripheral CD4 T Cells of Individuals on Successful Antiretroviral Therapy

**DOI:** 10.1128/mBio.03078-21

**Published:** 2021-11-30

**Authors:** Cristina Gálvez, Judith Grau-Expósito, Victor Urrea, Bonaventura Clotet, Vicenç Falcó, Maria José Buzón, Javier Martinez-Picado

**Affiliations:** a IrsiCaixa AIDS Research Institute, Badalona, Spain; b Hospital Universitari Vall d’Hebrón, Institut de Recerca (VHIR), Barcelona, Spain; c University of Vic-Central University of Catalonia (UVic-UCC), Vic, Spain; d Fundació Lluita contra la SIDA, Badalona, Spain; e Institute for Health Science Research Germans Trias i Pujol (IGTP), Badalona, Spain; f CIBER en Enfermedades Infecciosas, Madrid, Spain; g Catalan Institution for Research and Advanced Studies (ICREA), Barcelona, Spain; University of North Carolina at Chapel Hill; Albert Einstein College of Medicine

**Keywords:** HIV-1, HIV-1 DNA, HIV-1 RNA, HIV-1 cure, HIV-1 reservoir, HIV-1 reservoir size

## Abstract

Knowing the mechanisms that govern the persistence of infected CD4^+^ subpopulations could help us to design new therapies to cure HIV-1 infection. We evaluated the simultaneous distribution of the HIV-1 reservoir in 13 CD4^+^ subpopulations from 14 HIV-1-infected individuals on antiretroviral therapy to analyze its relationship with HIV-1 transcription, immune activation, and cell proliferation. A unique large blood donation was used to isolate CD4, CD4 resting (CD4r), CD4 activated (CD4a), T naive (T_N_), T stem cell memory (T_SCM_), T central memory (T_CM_), T transitional memory (T_TM_), T effector memory (T_EM_), circulating T follicular helper (_c_T_FH_), T_CD20_, T_CD32_, and resting memory T_CD2_^high^ (_rm_T_CD2_^high^) cells. HIV-1 DNA measured by droplet digital PCR ranged from 3,636 copies/10^6^ in T_TM_ to 244 in peripheral blood mononuclear cells (PBMCs), with no subpopulation standing out for provirus enrichment. Importantly, all the subpopulations harbored intact provirus by intact provirus DNA assay (IPDA). T_CD32_, _c_T_FH_, and T_TM_ had the highest levels of HIV-1 transcription measured by fluorescent *in situ* hybridization with flow cytometry (FISH/flow), but without reaching statistical differences. The subpopulations more enriched in provirus had a memory phenotype, were less activated (measured by CD38^+^/HLA-DR^+^), and expressed more programmed cell death 1 (PD-1). Conversely, subpopulations transcribing more HIV-1 RNA were not necessarily enriched in provirus and were more activated (measured by CD38^+^/HLA-DR^+^) and more proliferative (measured by Ki-67). In conclusion, the HIV reservoir is composed of a mosaic of subpopulations contributing to the HIV-1 persistence through different mechanisms such as susceptibility to infection, provirus intactness, or transcriptional status. The narrow range of reservoir differences between the different blood cell subsets tested suggests limited efficacy in targeting only specific cell subpopulations during HIV-1 cure strategies.

## INTRODUCTION

Antiretroviral therapy (ART) effectively blocks HIV-1 replication and reduces circulating virus to undetectable levels. However, ART is not able to eradicate the virus, and viral load rebounds rapidly if it is interrupted (1). Viral rebound results from the persistence of HIV-1, mostly in long-lived CD4^+^ T cells infected with integrated latent virus, which is the main obstacle to eradication of HIV-1 (2).

Numerous strategies are currently being evaluated to target and kill HIV-1-infected cells, with the ultimate goal of eradicating the virus (3). However, major obstacles include the inability to distinguish between latently infected cells *in vivo* and to ascertain their mechanisms of persistence. Therefore, it is of great interest to define which cell subpopulations compose the HIV-1 reservoir and their specific mechanisms of persistence in order to design effective therapies to cure HIV-1 infection.

To date, various cell subpopulations and cell markers have been identified as important HIV-1 reservoir cells. Central memory T cells (T_CM_) were reported to be a significant source of infected cells among CD4^+^ T-memory cells (4). Years later, stem cell memory T cells (T_SCM_) (5) were also postulated as a major cell subpopulation, since their long life makes them a main target for eradication strategies (6), despite their relatively low net contribution to the total reservoir. Recent studies have postulated CD4^+^ T follicular helper cells (T_FH_) (7), resting memory T_CD2_^high^ (_rm_T_CD2_^high^) (8), T_CD32_ (9, 10), and T_CD20_ (11) as important candidates. However, the set of cell subpopulations defined by these potential markers of viral persistence has never been studied simultaneously in the same individuals with the aim of defining more accurately their relative contribution to the structure of the viral reservoir in peripheral blood.

To address this issue, we comprehensively evaluated the distribution of the HIV-1 reservoir in the above-mentioned CD4^+^ T-cell subpopulations in peripheral blood and their relationship with RNA expression, immune activation, cell proliferation, and cytokine profiling.

## RESULTS

### Participants and study design.

**FIG 1:**
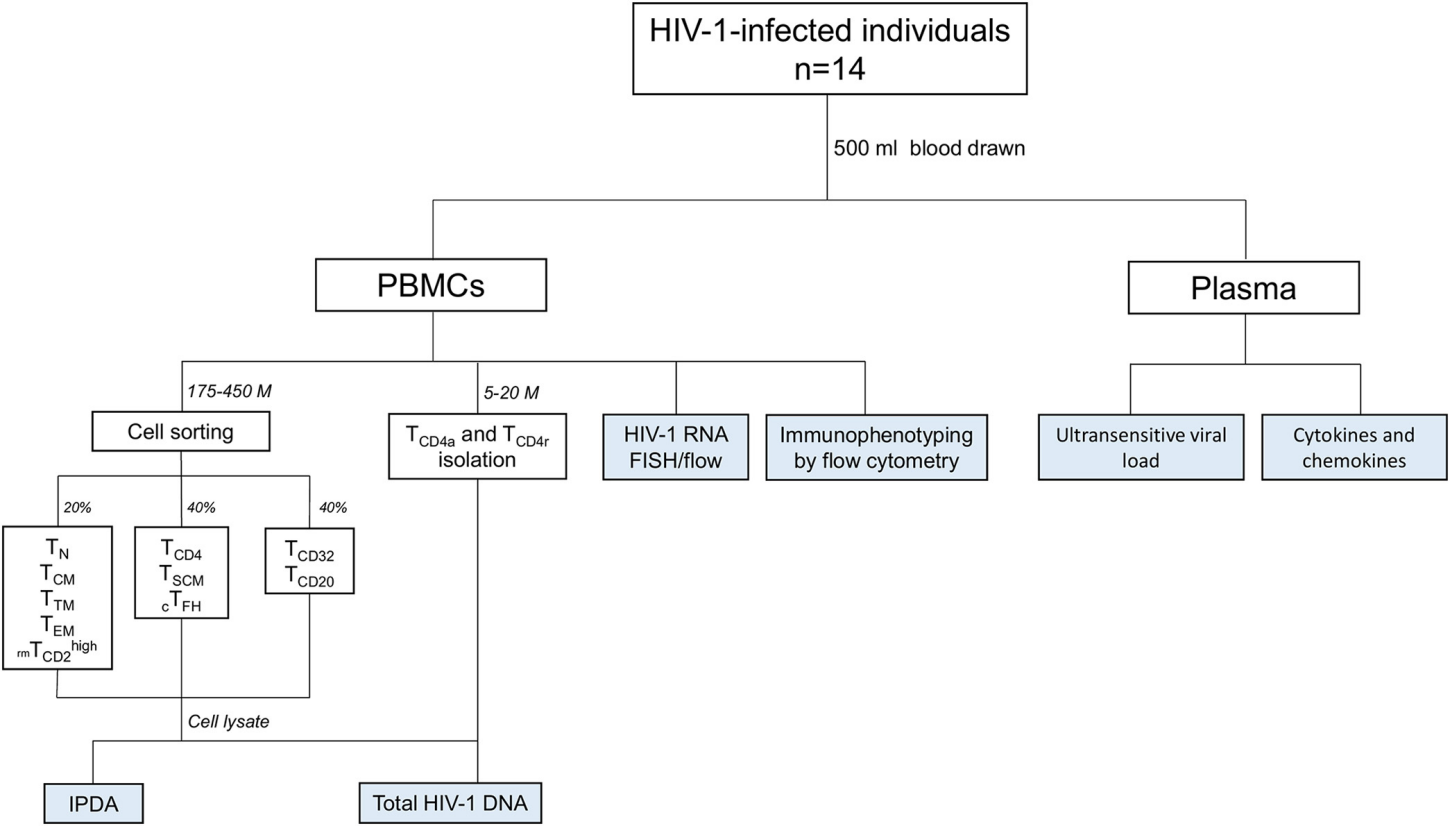
Study design flow-chart.

We studied 14 virologically suppressed HIV-1-infected individuals to assess the contribution of the CD4^+^ T-cell subpopulations to the viral reservoir. Their clinical characteristics are summarized in Table 1. All participants had been infected for more than 3 years when samples were taken, had CD4 counts over 400 cells/μL, and had undetectable viral loads for more than 2.8 years (<50 copies/mL plasma). A 500-mL blood sample was drawn to obtain peripheral blood mononuclear cells (PBMCs) and plasma. PBMCs were used for both immunomagnetic and fluorescence-activated cell sorting of the different CD4^+^ T-cell subpopulations of interest in order to measure the HIV-1 reservoir (total or intact), intracellular HIV-1 RNA expression, cell activation, programmed cell death 1 (PD-1) expression, and cell proliferation. Plasma was used to measure residual viremia by ultrasensitive viral load (usVL) and cytokines/chemokines in plasma (Fig. 1).

**TABLE 1:** Clinical characteristics of the subjects included in the longitudinal study^*a*^

Patient ID	Gender	Route of infection	Time since HIV-1 diagnosis (yrs)	Time with undetectable viral load (yrs)	Minimum CD4 cell count reported	Maximum viral load reported	CD4 cell count at sample (cells/μL)	usVL at sample (copies/mL)	ART at sample
1	Male	MSM/BI	16.6	10.3	382	640,000	1,200	<0.5	3TC, ABC, DTG
2	Male	HTSX	20.0	9.3	5	71,550	400	<0.5	3TC, ABC, DTG
3	Male	IVDU	21.0	7.5	310	450,000	990	1.7	COB, DRV
4	Male	MSM/BI	18.7	16.2	48	1,000,000	890	1.6	COB, EVG, FTC, TAF
5	Male	MSM/BI	9.4	4.3	536	830,000	1,110	2.9	FTC, RPV, TDF
6	Male	MSM/BI	15.1	14.8	20	460,000	760	14.0	COB, FTC, TAF, EVG
7	Male	HTSX	28.8	15.8	64	1,300,000	590	3.1	COB, DRV, ETV
8	Male	HTSX	5.3	3.4	45	910,000	610	2.2	DTG, RPV
9	Male	HTSX	11.8	11.7	129	19,000	530	0.6	COB, EVG, FTC, TAF
10	Female	IVDU	28.0	7.9	36	3,630,000	810	7.9	COB, EVG, FTC, TAF
11	Male	HTSX	28.1	6.4	302	170,000	740	<0.5	EFV, FTC, TDF
12	Male	MSM/BI	3.0	2.8	540	287,000	910	<0.5	COB, FTC, TAF, EVG
13	Male	IVDU	24.6	6.0	280	23,000	720	1.1	TDF, COB, DRV
14	Male	MSM/BI	12.3	8.0	400	550,000	1,310	2.4	EFV, FTC, TDF

a MSM, men who have sex with men; BI, bisexual; HTSX, heterosexual; IVDU, intravenous drug users; usVL, ultrasensitive viral load; ART, antiretroviral therapy; 3TC, lamivudine; ABC, abacavir; DTG, dolutegravir; COB, cobicistat; DRV, darunavir; EVG, elvitegravir; FTC, emtricitabine; TAF, tenofovir alafenamide; RPV, rilpivirine; TDF, tenofovir disoproxil fumarate; ETV, etravirine.

### Determination of HIV-1 DNA in CD4^+^ T-cell subpopulations.

**FIG 2:**
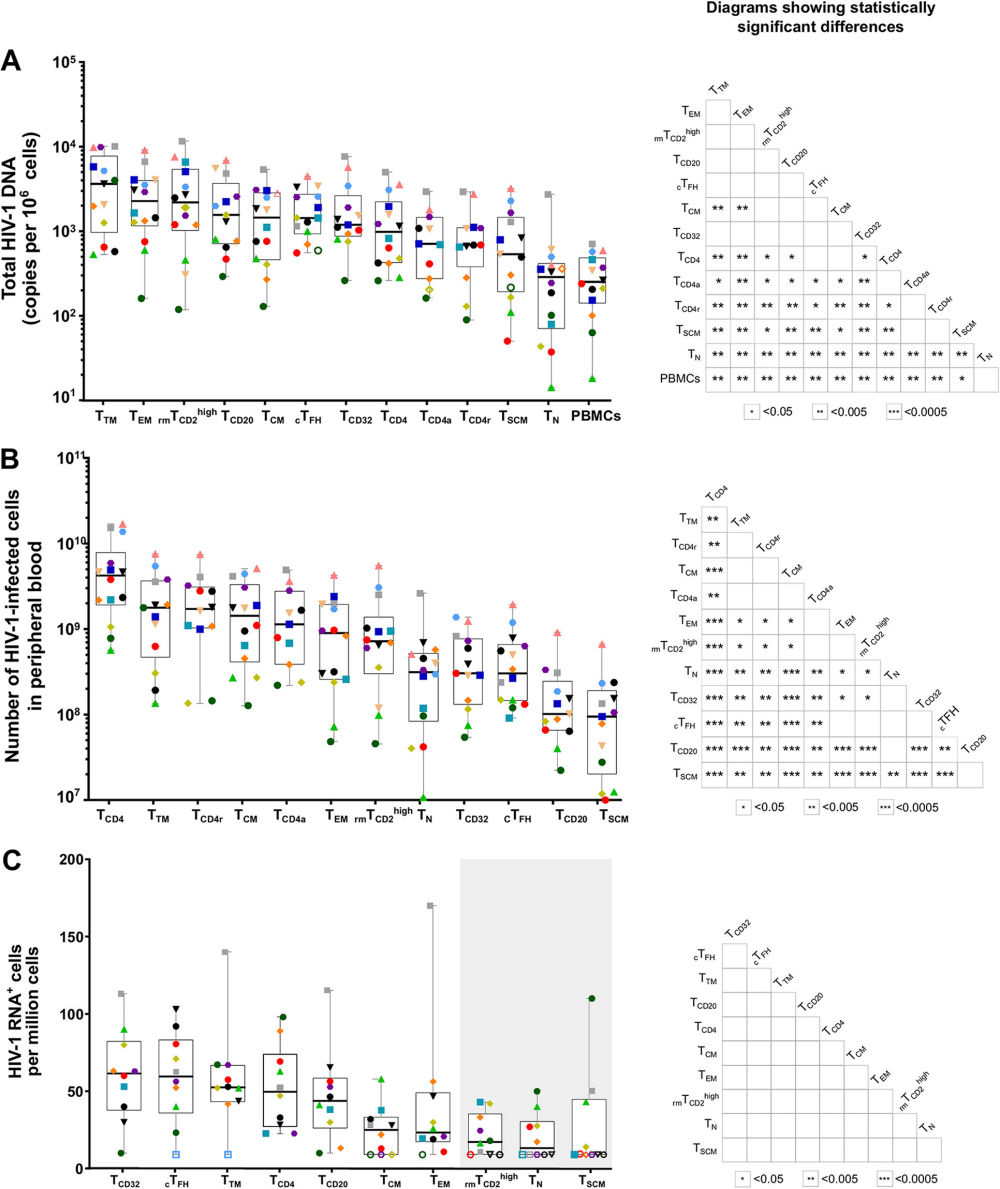
Measurement of HIV-1 DNA and HIV-1 RNA in CD4^+^ T-cell subpopulations. (A) Total HIV-1 DNA measured in 13 sorted CD4^+^ T-cell subpopulations. According to the gating strategy (Fig. S1), some of these subpopulations overlap. (B) Number of HIV-1-infected cells for each CD4^+^ T-cell subpopulation based on the frequency of each CD4^+^ T-cell subpopulation and its levels of total HIV-1 DNA in peripheral blood. (C) Proportion of cells expressing HIV-1 RNA in each CD4^+^ T-cell subpopulation normalized to the medium control. The gray box indicates the cell subpopulations with median HIV-1^+^ RNA levels not different from HIV-1^–^ individuals. (A to C) For all the panels, each colored symbol represents a single individual. On the right of each panel, matrices depict *P* values of the differences between paired CD4^+^ T-cell subpopulations. Differences were tested for statistical significance using the Wilcoxon rank sum test followed by the FDR test for multiple comparisons. Box and whisker plots indicate the median, interquartile range, and minimum and maximum values.

In order to determine whether the stability of infected cells could be attributed to specific CD4^+^ T-cell subpopulations, we sorted CD4^+^ T-cell subpopulations (Fig. S1; Table 2) from successfully virologically suppressed HIV-1-infected individuals based on expression of multiple surface markers and quantified total HIV-1 DNA in each subpopulation using droplet digital PCR (ddPCR). Median total HIV-1 DNA was detected in all cell subpopulations analyzed, gradually ranging from 3,636 to 244 copies per million cells of a given subset (Fig. 2A).

**TABLE 2:** Number of cells sorted for each cell subpopulation

Cell subpopulation	Median^*a*^	Minimum	Maximum
T_CD4_	842,857	500,000	1,000,000
T_CD4r_	1,532,367	540,400	3,000,000
T_CD4a_	1,036,150	554,800	2,230,000
T_N_	535,246	126,000	1,450,000
T_CM_	475,761	89,000	835,000
T_TM_	55,115	4,400	141,216
T_EM_	454,623	66,000	753,171
_rm_T_CD2_^high^	271,786	55,000	500,000
T_SCM_	106,419	11,866	277,623
_c_T_FH_	263,779	19,600	507,000
T_CD32_	113,782	26,000	318,000
T_CD20_	334,635	118,000	435,000

a Cells were equally distributed to measure total HIV-1 DNA and IPDA.

10.1128/mBio.03078-21.1 FIG S1 A representative gating strategy used for cell sorting of the cell subpopulations. Three separate cell sorters were made in order to be able to sort the overlapping subpopulations. A common gate strategy was used where PBMCs were acquired and gated according to (i) forward scatter area (FSC-A) and forward scatter height (FSC-H) to select singlets, (ii) side scatter area (SSC-A) and FSC-A to determine cell morphology, (iii) CD3 and viability staining to select living T cells, and (iv) CD4 T cells from viable cells to gate all the other cell subpopulations. In sorter 1, first, a fraction of _rm_T_CD2_^high^ were sorted because of overlap with all the other cell subpopulations. Then, T_N_, T_CM_, T_EM_, and T_TM_ were discriminated with CD45RA, CCR7, and CD27 markers as described in Materials and Methods. In sorter 2, T_SCM_ and _c_T_FH_ were sorted from CD4^+^ T cells. Finally, T_CD32_ and T_CD20_ were sorted in sorter 3. Download FIG S1, TIF file, 0.8 MB. Copyright © 2021 Gálvez et al. 2021 Gálvez et al. https://creativecommons.org/licenses/by/4.0/ This content is distributed under the terms of the Creative Commons Attribution 4.0 International license.

We observed that the most infected cell subpopulations had a memory phenotype, namely, T transitional memory (T_TM_), T effector memory (T_EM_), _rm_T_CD2_^high^, T_CD20_, and T_CM_ cells (Fig. 2A). Statistically significant differences were found between multiple cell subpopulations (see *P* value matrices in Fig. 2A). However, although the distribution of total HIV-1 DNA gradually decreased across the CD4^+^ T-cell populations studied to the naive phenotype (T_N_) and total PBMCs, no specific subpopulation was noteworthy for enrichment in provirus. In addition, we calculated the total number of infected cells in peripheral blood for each subpopulation by considering the number of CD4^+^ T cells per μL in peripheral blood per individual, an assumed mean blood volume of 5 L per person, and the percentage of each subpopulation among CD4^+^ T cells obtained by flow cytometry (Fig. 2B). We observed a higher number of infected T_TM_ and CD4 resting (CD4r), followed by T_CM_, CD4 activated (CD4a), and T_EM_. At the other end of the spectrum, the total number of HIV-1-infected cells in the peripheral blood of subpopulations, such as T_CD20_ and T_SCM_, was minimal.

The contribution of each subpopulation to the total HIV-1 reservoir could not be calculated, owing to the unavoidable overlap between specific cell subpopulations. Nevertheless, we calculated the contribution of the nonoverlapping T_N_, T_SCM_, T_CM_, T_TM_, and T_EM_ cells to the HIV-1 reservoir and found the highest values for T_CM_ cells (median contribution of 37%), followed by T_TM_ cells (32%) and T_EM_ cells (23%), whereas the contribution of T_N_ cells and T_SCM_ cells to the pool of cells harboring HIV-1 proviral DNA was marginal (8% and 0.2%, respectively) (Fig. S2A).

10.1128/mBio.03078-21.2 FIG S2 Contribution of memory cell subpopulations and HIV/RNA ratio. (A) Contribution of the T_N_, T_SCM_, T_CM_, T_TM_, and T_EM_ cell subpopulations to the pool of HIV-infected cells in 14 HIV-infected individuals. Differences were tested for statistical significance using Friedman’s test followed by the false-discovery rate test for multiple comparisons. (B) Ratio between HIV-1 RNA and HIV-1 DNA. Each symbol represents a different individual. RNA/DNA ratios for T_N_, T_SCM_, and _rm_T_CD2_^high^ were not represented due to the high number of undetectable values for intracellular HIV-1 RNA measurements. Box and whisker plots indicate the median, interquartile range, and minimum and maximum values. Download FIG S2, TIF file, 0.2 MB. Copyright © 2021 Gálvez et al. 2021 Gálvez et al. https://creativecommons.org/licenses/by/4.0/ This content is distributed under the terms of the Creative Commons Attribution 4.0 International license.

Moreover, we analyzed the correlations between total HIV-1 DNA in each CD4^+^ T-cell subpopulation and clinical parameters (Table S1). HIV-1 DNA was positively and significatively correlated with years with undetectable viral load in circulating T follicular helper (_c_T_FH_) (rho = 0.56, *P* = 0.04) and also with maximum viral load reported with T_CD32_ (rho = 0.58, *P* = 0.04). However, the significance of these correlations did not hold after adjusting for multiple comparisons.

10.1128/mBio.03078-21.8 TABLE S1 Analysis of the correlation between total HIV-1 DNA, clinical parameters, HIV-1 expression, and cytokines/chemokines plasma levels. Download Table S1, DOCX file, 0.02 MB. Copyright © 2021 Gálvez et al. 2021 Gálvez et al. https://creativecommons.org/licenses/by/4.0/ This content is distributed under the terms of the Creative Commons Attribution 4.0 International license.

While subpopulations with a memory phenotype are generally richer in provirus, HIV-1 infection does not seem to occur preferentially in a very specific cell subpopulation, thus demonstrating the high heterogeneity of the HIV-1 reservoir.

### Determination of intracellular HIV-1 RNA in CD4^+^ T-cell subpopulations.

Since most HIV-1 DNA is defective, we sought to investigate the transcriptional activity of HIV-1 in subpopulations from 10 HIV-1-infected individuals (Fig. S4). We used the novel RNA fluorescent *in situ* hybridization with flow cytometry (FISH/flow) protocol to measure the frequency of each subpopulation expressing intracellular HIV-1 RNA (12) and found that T_CD32_, _c_T_FH_, T_TM_, and T_CD20_ were the cell populations with higher HIV-1 RNA transcription (median values in HIV-1 RNA^+^ cells per million CD4^+^ T cells: T_CD32_, 61.5; _c_T_FH_, 59.5; T_TM_, 52.5; and T_CD20_ 43.8). Subpopulations such as _rm_T_CD2_^high^, T_N_, and T_SCM_ barely expressed HIV-1 viral transcripts, since their median levels were not different from 0 after background subtraction (Fig. 2C and Fig. S5). Nonetheless, differences between the cell subpopulations were not statistically significant, probably due to the lower number of samples assayed. We also showed that most HIV-1 DNA was not transcribed, as revealed by the HIV-1 RNA/DNA ratio, which was lower than 0.05 in all the CD4^+^ T-cell subpopulations analyzed (Fig. S2B).

10.1128/mBio.03078-21.4 FIG S4 Representative plots of HIV-1 RNA expressing cells. Levels of intracellular HIV-1 RNA in T_TM_ cells from an uninfected donor, which is used to set up the limit of detection of the assay, and two individuals from the study. Download FIG S4, TIF file, 0.7 MB. Copyright © 2021 Gálvez et al. 2021 Gálvez et al. https://creativecommons.org/licenses/by/4.0/ This content is distributed under the terms of the Creative Commons Attribution 4.0 International license.

10.1128/mBio.03078-21.5 FIG S5 Comparison of levels of HIV-1 RNA expressing cells between HIV-1^–^ and HIV-1^+^ individuals. Differences were tested for statistical significance using the Wilcoxon matched-pair rank test. Download FIG S5, TIF file, 0.5 MB. Copyright © 2021 Gálvez et al. 2021 Gálvez et al. https://creativecommons.org/licenses/by/4.0/ This content is distributed under the terms of the Creative Commons Attribution 4.0 International license.

Levels of intracellular HIV-1 expression were correlated with total HIV-1 DNA in T_TM_ (rho = 0.7, *P* = 0.04) and T_CD4_ cells (rho = −0.66, *P* = 0.04) (Table S1). Additionally, we measured HIV-1 expression in plasma by usVL and found statistically significant positive correlations between total HIV-1 DNA and usVL in 6 subpopulations (Table S1), namely, T_SCM_ (rho = 0.71, *P* = 0.01), T_EM_ (rho = 0.7, *P* = 0.01), _c_T_FH_ (rho = 0.7, *P* = 0.01), T_CD32_ (rho = 0.69, *P* = 0.01), T_N_ (rho = 0.66, *P* = 0.01), and T_CD20_ (rho = 0.57, *P* = 0.04). However, after adjusting for multiple comparisons, the significance of the correlations did not hold.

Thus, these results suggest that the cells most enriched in provirus are not the most transcriptionally active ones.

### Quantification of intact provirus in CD4^+^ T-cell subpopulations.

**FIG 3:**
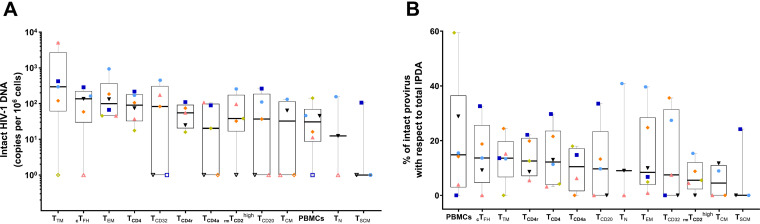
Measurement of intact provirus in CD4^+^ T-cell subpopulations. (A) Intact proviral frequencies per 10^6^ cells determined by IPDA for each CD4^+^ T-cell subpopulation from 6 HIV-1-infected individuals. (B) Percentage of intact provirus with respect to total IPDA levels. Total IPDA proviruses were determined as the sum of intact, 5′-defective, and 3′-defective proviruses from each individual. Each symbol represents 1 individual. Open symbols represent values under the limit of detection. Box and whisker plots indicate the median, interquartile range, and minimum and maximum values.

To assess the number of intact viruses in each cell subpopulation, we performed the intact provirus DNA assay (IPDA) in PBMCs from the 14 HIV-1-infected individuals. In 6 individuals, we performed IPDA in all the cell subpopulations based on sample availability. We observed that all subpopulations measured had intact provirus to a different extent, with the most transcriptionally active cells, such as T_TM_ and _c_T_FH_, being the ones with the most intact provirus (Fig. 3A); nonetheless, there were not statistically significant differences. Of note, T_TM_ was the subpopulation with the highest total HIV-1 DNA (Fig. 2A) and intact provirus (Fig. 3A). Moreover, the proportion of intact provirus with respect to total IPDA was generally low, with the highest median values found in PBMCs (14.8%), _c_T_FH_ (13.6%), and T_TM_ (13.6%) and the lowest in T_CM_ (4.5%) and T_SCM_ (0%) (Fig. 3B).

### Relationship between the parameters analyzed in each CD4^+^ T-cell subpopulation.

We then analyzed the relative proportion of HIV-1 DNA, HIV-1 RNA, activation, proliferation, and PD-1 markers between the different subpopulations (Fig. S6). We included CD69 for early activation, HLA-DR for late activation, and double-positive cells for CD38 and HLA-DR (Fig. S7A to C). Also, we used Ki-67 as a proliferation maker, and PD-1 as a surrogate marker of HIV latency promoter (Fig. S7D and E).

10.1128/mBio.03078-21.6 FIG S6 Representative example of the gating strategy used for analyses in Fig. S7A to E. PBMCs were acquired and gated according to (i) time to ensure homogeneous acquisition, (ii) SSC-A and FSC-A to determine cell morphology, (iii) FSC-A and FSC-H to select singlets, (iv) CD3 and viability staining to select living T cells, and (v) CD4 T cells from viable cells to gate all the other cell subpopulations. Then CD69, CD38, HLA-DR, PD-1, and CD38^+^HLADR^+^ were gated on every cell subpopulation based on FMOs. The graph shows a representative gate strategy for CD4^+^ T cells, but the same was made for T_N_, T_SCM_, T_CM_, T_TM_, T_EM_, _c_T_FH_, T_CD20_, T_CD32_, and _rm_T_CD2_^high^ that were gated as in Fig. S1. Download FIG S6, TIF file, 0.7 MB. Copyright © 2021 Gálvez et al. 2021 Gálvez et al. https://creativecommons.org/licenses/by/4.0/ This content is distributed under the terms of the Creative Commons Attribution 4.0 International license.

10.1128/mBio.03078-21.7 FIG S7 Analysis of activation, proliferation, and exhaustion in CD4^+^ T-cell subpopulations. (A to C) Percentage of activation levels based on (A) CD69^+^, (B) HLA-DR^+^, and (C) double positive CD38^+^ and HLA-DR^+^ cells for each CD4^+^ T-cell subpopulation. (D) Percentage of cell proliferation measured using Ki-67^+^ cells and (E) percentage of PD-1^+^ cells. Each symbol represents 1 individual. (A to E) Matrices depict *P* values of the differences between paired CD4^+^ T-cell subpopulations. Differences were tested for statistical significance using the Wilcoxon rank sum test followed by the false-discovery rate test for multiple comparisons. Box and whisker plots indicate the median, interquartile range, and minimum and maximum values. Download FIG S7, TIF file, 0.6 MB. Copyright © 2021 Gálvez et al. 2021 Gálvez et al. https://creativecommons.org/licenses/by/4.0/ This content is distributed under the terms of the Creative Commons Attribution 4.0 International license.

**FIG 4:**
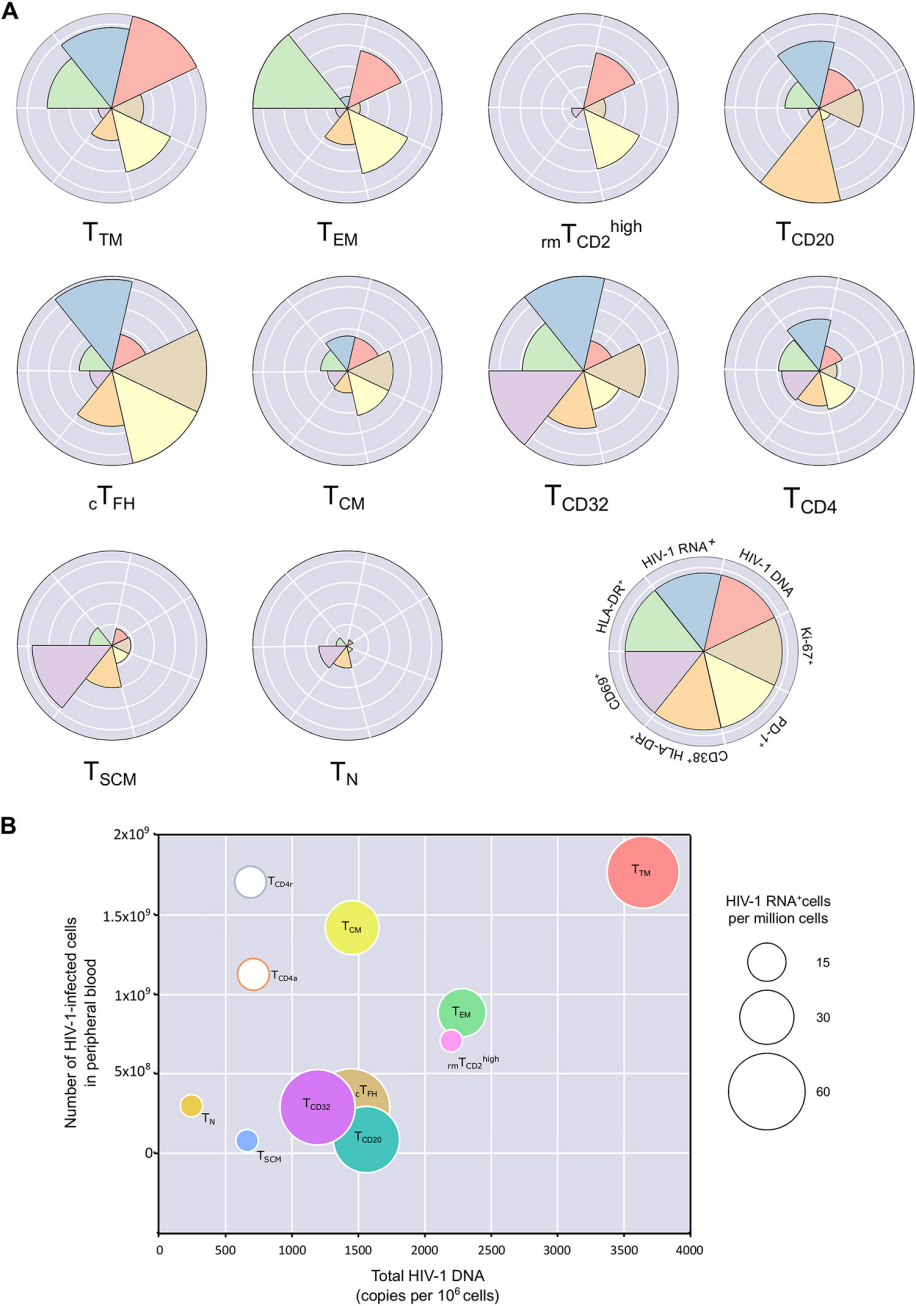
Relationship between HIV-1 DNA, HIV-1 RNA, and parameters of activation, exhaustion, and proliferation for each CD4^+^ T-cell subpopulation. (A) The Nightingale rose plots show the patterns of 7 parameters (total HIV-1 DNA, HIV-1 RNA^+^, Ki-67^+^, PD1^+^, CD38^+^HLA-DR^+^, CD69^+^, HLA-DR^+^) for 10 CD4^+^ T-cell subpopulations. Each wedge represents a parameter studied and is depicted in a different color. The size of the wedge for each parameter depicts a proportional magnitude of the parameter relative to the maximum magnitude across the different CD4^+^ T-cell subpopulations. (B) Three-dimensional graph showing the correlation between total HIV-1 DNA and the number of HIV-1-infected cells in peripheral blood; the bubble size represents the levels of intracellular HIV-1 RNA in each cell subpopulation. T_CD4r_ and T_CD4a_ bubbles are not sized according to their intracellular HIV-1 RNA. T_N_, T_SCM_, and _rm_T_CD2_^high^ are given a small bubble size since their intracellular HIV-1 RNA levels are not different from those of HIV-1^–^ individuals.

We observed that the subpopulations with the highest total HIV-1 DNA value, with the exception of T_CD20_, had higher expression of PD-1, which has been described as actively promoting HIV latency (13, 14) (Fig. 4A and Fig. S7E). It is noteworthy that these subpopulations were those with a more differentiated phenotype, such as T_EM_ and T_TM_, while the less differentiated cells and those with less provirus (T_N_ and T_SCM_) generally had very low frequencies of PD-1 (Fig. 4A and Fig. S7E). Similarly, the activation profile based on the double expression of CD38/HLA-DR (Fig. S7C) showed that cell subpopulations with a larger reservoir were not highly activated (Fig. 4A). Regarding proliferation, the cell subpopulations with the highest levels of Ki-67 were those that also had the highest levels of HIV-1 RNA expression, such as _c_T_FH_ and T_CD32_ (Fig. S7D). Interestingly, T_CD32_ cells (and to a lesser extent total CD4^+^ T cells) had average total HIV-1 DNA but high HIV-1 expression, activation, and proliferation, thus indicating a nonquiescent state (Fig. 4A). In contrast, subpopulations with the lowest levels of total HIV-1 DNA (T_N_ and T_SCM_) expressed less PD-1 and were nonproliferating and did not express HIV-1 RNA (Fig. 4A).

We performed a more in-depth analysis of the relationship between total HIV-1 DNA per CD4 T-cell subpopulation, the number of HIV-1-infected cells in total peripheral blood, and the expression of intracellular HIV-1 RNA measured by FISH/flow in each cell subpopulation (Fig. 4B). With the exception of T_CD4r_ and T_CD4a_ cell populations, most of the CD4^+^ T-cell subpopulations showed a certain degree of correlation between total HIV-1 DNA per CD4^+^ T-cell subpopulation and the number of HIV-1-infected cells in total peripheral blood. However, expression of intracellular HIV-1 RNA varied more widely between cell subpopulations. Thus, T_TM_ was the most infected CD4^+^ T-cell subpopulation, the most abundant in peripheral blood, and one of the most highly transcriptionally active (Fig. 4B). The most transcriptionally active remaining cell subpopulations (T_CD32_, _c_T_FH_, T_CD20_) clustered together with intermediate levels of HIV-1 DNA and an average number of infected cells in total peripheral blood (Fig. 4B).

Overall, these results indicate that the transcriptionally inactive HIV-1 reservoir is composed mainly of cells with low levels of activation, high levels of PD-1, and low proliferation, potentially indicating the quiescent nonactivated state of the cells that constitute the HIV-1 reservoir in individuals receiving ART.

### Cytokines and chemokines in plasma.

To further characterize how residual inflammation might affect the composition of the viral CD4^+^ T-cell reservoir, we measured 10 cytokines/chemokines using Luminex in the plasma of 14 ART-treated individuals. We found statistically significant negative correlations between interferon gamma (IFN-γ) and total HIV-1 DNA in T_CD32, c_T_FH_, and T_SCM_ (Table S1), as well as between tumor necrosis factor (TNF) and T_CM_, T_CD32_, and T_CD4_. Moreover, statistically significant negative correlations were observed for the cytokine interleukin 17A (IL-17A)/cytotoxic-T-lymphocyte-associated antigen 8 (CTLA8) and total HIV-1 DNA in 4 out of 10 subpopulations studied, including T_TM_, _rm_T_CD2_^high^, T_CM_, and T_SCM_ (Table S1). Nonetheless, correlations did not hold after false-discovery rate (FDR) adjustment. These results suggest a subtle link between levels of HIV-1 reservoir in the different cell subpopulations and the inflammation profile.

## DISCUSSION

Elimination of HIV-1-infected cells is one of the current priorities in HIV-1 research (15). However, we are still unable to identify infected cells *in vivo* that can be specifically targeted in therapeutic strategies. In this study, we simultaneously and comprehensively analyzed proviral DNA levels, transcription, activation status, and inflammatory profile in up to 13 relevant CD4 T-cell subsets from the same blood samples of a unique cohort of people with HIV who were on suppressive antiretroviral therapy. Therefore, this study helps to establish a more precise comparison among previously described viral reservoirs in blood cells, diminishing the effect of patient, sample, and technique variability.

First, we observed that total HIV-1 DNA content was always present and gradually distributed in all the CD4^+^ T-cell subpopulations analyzed, with a maximum variation of the medians of about 1 log. No specific subpopulation stood out from the rest regarding enrichment in provirus, and therefore targeting a specific subpopulation in HIV eradication strategies will result in limited changes of the total reservoir size. However, as previously described, cell subpopulations with a memory phenotype, such as T_TM_, T_EM_, and _rm_T_CD2_^high^, were more enriched in provirus, while T_N_ cells harbored the smallest reservoir (4). Thus, driving the HIV-1-infected cells toward terminal effector cell subpopulations that have shorter half-lives might help to diminish the viral reservoir, as recently suggested by Grossman et al. (16).

Nevertheless, to fully estimate the final contribution of the viral reservoir in peripheral blood, the size of each cell subpopulation must also be taken into account. In this regard, T_TM_, T_CD4r_, and T_CM_ subpopulations were the main contributors to the HIV reservoir in peripheral blood. This is probably the result of the long half-life and self-renewal capacities that characterize the memory subsets (17, 18).

Another important factor to consider is the intactness of the provirus, since only intact proviruses are likely to be infectious and therefore contribute to viral rebound if the pressure of ART is interrupted (19). Importantly, we detected intact provirus measured by IPDA in all the cell subpopulations analyzed in the subset of individuals whose samples were available, suggesting that all the infected cell subpopulations might be a source of viral rebound, which would be in line with previous reports (20) In all cases, however, intact provirus represented less than 15% of the total HIV-1 DNA, as previously reported (21). Although there were not statistically significant differences, likely due to the small sample size, the results suggested that T_TM_, _c_T_FH_, T_EM_, and T_CD32_ were slightly more enriched in intact provirus.

Latently infected cells in individuals on ART are generally not transcriptionally active (22), although this observation might differ depending on the cell type, owing to differences in activation state or the proportion of defective provirus. We found that cell subpopulations with higher intact provirus, in general, expressed more intracellular HIV-1 RNA as measured by FISH/flow, including T_CD32_, _c_T_FH_, and T_TM_, indicating that it would be important to tackle not only cells in a resting state, but also the active viral reservoir, since these might be sources of residual viremia during ART and viral rebound if ART is discontinued. In fact, a recent study showed that viral RNA expression after latency reversal was detected in all CD4^+^ T-cell subpopulations assayed (23), indicating that when properly stimulated, most cells are able to sustain viral transcription.

We also considered whether there were differences in the activation, proliferation, and PD-1 levels between the different cell subpopulations. It is known that a larger fraction of the viral reservoir is mostly composed of resting and exhausted cells with low rates of proliferation (2, 24), a finding that is consistent with our results. Specifically, highly activated cell subpopulations measured by CD38^+^HLA-DR^+^, such as _c_T_FH_ and T_CD32_, were, as expected, highly transcriptionally active but not extraordinarily enriched in provirus, confirming previous findings on T_CD32_ cells (25, 26). On the other hand, the T_TM_, T_EM_, and _rm_T_CD2_^high^ subpopulations that harbored more provirus, were also the ones expressing more PD-1, probably due to the persistent exposure to HIV-1 antigens. The frequencies and distribution of CD4^+^ T cells expressing PD-1 were similar to those reported in the study from Fromentin et al. (24). Importantly, clonal proliferation of HIV-1-infected cells has been identified as a key mechanism of viral persistence *in vivo* (27, 28). In this regard, we found that some of the most transcriptionally active subsets (T_CD32_ and _c_T_FH_ cells) also have a higher cell proliferation capacity, as measured according to expression of Ki-67. The way in which these subpopulations might be associated with the clonal expansion of specific HIV-1-infected cells *in vivo* warrants further investigation.

Surprisingly, we found a negative correlation between levels of HIV-1 DNA in several cell subpopulations and the plasma cytokine IL-17. An association has been observed between high levels of plasma viremia and depletion of Th17 cells, which produce IL-17 (29). Although we did not study Th17 cells, the positive correlation found between usVL and levels of total HIV-1 DNA leads us to speculate that individuals with a larger HIV-1 reservoir might have a lower frequency of Th17 cells and, therefore, lower levels of IL-17 in plasma. Given the recent finding implicating Th17 cells as sanctuaries for HIV-1 reservoirs (30), viral persistence may modulate cell epigenetic states and signaling pathways to allow viral latency that can impair IL-17 release, thus rendering Th17 cells dysfunctional (31). Finally, as IL-17 contributes to the integrity of the gut barrier, low levels of Th17 may be associated with greater microbial translocation, which may increase residual inflammation and favor HIV-1 persistence. Nonetheless, these explanations are speculative, and further investigations should be performed to prove them.

Our findings indicate that the HIV-1 reservoir in peripheral blood is composed of a mosaic of HIV-1-infected cell subpopulations contributing to the persistence of HIV-1 through a series of mechanisms, including susceptibility to infection (T_TM_, T_EM_, _rm_T_CD2_^high^), higher rates of intact provirus (T_TM_, _c_T_FH_, T_EM_), transcriptionally active subpopulations (_c_T_FH_, T_CD32_), and subpopulations with a long half-life (T_SCM_, T_CM_) (5, 18). Therefore, these results suggest that eradication strategies relying solely upon targeting unique cell subpopulations would hardly be effective. In fact, a recent study where they did a phenotypic analysis of the CD4 T-cell reservoir using cytometry by time of flight (CyTOF), reached a similar conclusion that a single surface marker/cell subpopulation does not exist to target the entire HIV-1 reservoir at once (32). A combination of strategies targeting their different characteristics would be more desirable.

Our study was subject to a series of limitations. First, it was only performed in peripheral blood, while it is known that most infected cells are found in lymph nodes and gut-associated lymphoid tissue and that the frequencies of cell subpopulations in tissues might differ. However, given the low frequencies of some of the cell subpopulations in the study, it would have been extremely challenging to perform the same comparative analysis in other tissues. These same low frequencies can also limit, in some cases, the precision of quantification of total HIV-1 DNA and measurements of intact proviruses in blood. Also, the technique used for measuring HIV-1-expressing cells has a relatively high background in HIV-1^–^ individuals. Finally, we did not analyze the functionality of the different cell subpopulations, for example, by measuring the replication competence of the provirus.

In conclusion, we found that the HIV-1 reservoir under ART is distributed throughout all CD4 T-cell subpopulations and has heterogeneous levels of HIV-1 expression, activation, and proliferation and, thus, contributes to HIV-1 persistence through different mechanisms, such as susceptibility to infection, rates of provirus intactness, transcriptional status, and half-life. This research provides new knowledge on the composition of the HIV-1 reservoir suggesting that eradication strategies relying solely upon targeting unique cell subpopulations will result in limited changes of the total reservoir size.

## MATERIALS AND METHODS

### Study participants.

Samples from 14 HIV-1-infected individuals on suppressive ART were obtained from the HIV unit of Hospital Universitari Vall d’Hebron in Barcelona, Spain. A 500-mL sample of blood was extracted to collect large numbers of peripheral blood mononuclear cells (PBMCs) and plasma. The local ethics committee approved the protocol [PR(AG)192/2018], and the study was performed in accordance with the principles of the Declaration of Helsinki. All participants provided written informed consent before initiation. Samples were obtained only from adults and were totally anonymous and untraceable.

### Cell sorting.

PBMCs were isolated from fresh blood (14 individuals) using Ficoll-Paque centrifugation. Resting and activated CD4^+^ T cells (CD4r and CD4a, respectively) were isolated from PBMCs using magnetic beads (MACS; Miltenyi Biotec) based on CD69, CD25, and HLA-DR activation markers. Then, starting from 175 × 10^6^ to 450 × 10^6^ PBMCs, other CD4^+^ T-cell subpopulations were sorted on a FACSAria II device (BD Biosciences) using 3 different sorting panels because of the overlap between the cell subpopulations (see sorting strategy in Fig. S1). PBMCs were incubated with the following series of antibodies: live/dead (allophycocyanin Cy7 [APC-Cy7]; Invitrogen), CD3 (BV510; BD Biosciences), CD4 (AF700; BD Biosciences), CD45RA (APC; BioLegend), CCR7 (PE Dazzle; BioLegend), CD27 (fluorescein isothiocyanate [FITC]; BD Biosciences), CD95 (phycoerythrin Cy5 [PE-cy5]; BD Biosciences), CXCR5 (PE-Cy7; BD Biosciences), PD-1 (BV421; BioLegend), HLA-DR (BV421; BD Biosciences), CD2 (BV605; BD Biosciences), CD32 (phycoerythrin [PE]; BioLegend), and CD20 (PerCPCy5.5; BD Biosciences). All sorted CD4^+^ T-cell subpopulations were defined as live CD3^+^CD4^+^ T cells. T-cell maturation was based on the expression of the surface markers CD45RA, CCR7, CD27, and CD95 in order to define the following subpopulations: T naive (T_N_, CD45RA^+^CCR7^+^CD27^+^), T stem cell memory (T_SCM_, CD45RA^+^CCR7^+^CD27^+^CD95^+^), T central memory (T_CM_, CD45RA-CCR7^+^CD27^+^), T transitional memory (T_TM_, CD45RA^–^CCR7^–^CD27^+^), and T effector memory (T_EM_, CD45RA^–^CCR7^–^CD27^–^). The remaining CD4^+^ T-cell subpopulations were circulating T follicular helper (_c_T_FH_; CXCR5^+^, PD1^+^), T_CD32_ (CD32^+^), T_CD20_ (CD20^+^), and _rm_T_CD2_^high^ (CD45RA^–^, HLA-DR^–^, CD2^high^) cells.

### Flow cytometry.

For phenotypic analysis, we stained PBMCs with the following series of antibodies: live/dead (APC-Cy7; Invitrogen), CD3 (APC-CY7; BioLegend), CD4 (AF700; BD Biosciences), CD2 (BV605; BD Biosciences), CD32 (PE; BioLegend), CD45RA (BV510; BioLegend), CCR7 (PE Dazzle; BioLegend), CD27 (FITC; BD Biosciences), CD95 (PE-Cy5; BD Biosciences), CXCR5 (PE Dazzle; BioLegend), PD-1 (BV421; BioLegend), HLA-DR (BV786; BD Biosciences), CD38 (BV711; BioLegend), CD20 (BV570; BioLegend), and CD69 APC; BD Biosciences). We ran the assays on an LSR Fortessa device (BD Biosciences), and data were analyzed using FlowJo software v10. Cutoffs for continuous markers were based on fluorescence-minus-one (FMO) controls (Fig. S6).

### Total HIV-1 DNA quantification.

Total HIV-1 DNA was measured in cell lysates of each cell subpopulation isolated using droplet digital PCR (ddPCR), as previously described (33). Briefly, the 5′ long terminal repeat (*5′LTR*) region or *Gag* was amplified, and the *RPP30* housekeeping gene was quantified in parallel to normalize sample input. Raw ddPCR data were analyzed using the QX100 droplet reader and QuantaSoft v1.6 software (Bio-Rad).

Undetectable samples are expressed as the limit of detection, which varies between samples depending on cell input. PBMCs from HIV-negative donors were used as negative controls and assayed in each plate to set the positive/negative threshold for ddPCR analysis.

### RNA FISH/flow assay of single cells expressing HIV-1 RNA transcripts.

PBMCs from 10 ART-treated HIV-infected patients were obtained using Ficoll Hypaque, and CD4^+^ T cells were isolated by negative selection using magnetic beads (MagniSort human CD4^+^ T cell enrichment; eBioscience). Then, cells underwent the RNA FISH/flow protocol for the detection of HIV-1 mRNA transcripts following the manufacturer’s instructions (Human PrimeFlow RNA assay; eBioscience), with some modifications, as previously described (12). Briefly, freshly isolated CD4^+^ T cells were stained with antibodies against cell surface markers and viability dye. Cells were then fixed, permeabilized, and intracellularly stained. After an additional fixation step, cells were prepared for 3 h with hybridization probes at 40 ± 1°C using a high-sensitivity target-specific set of 50 probes spanning the whole Gag-Pol HIV mRNA sequence (bases 1165 to 4402 of the HIV-1_HXB2_ consensus genome). Subsequently, cells underwent various amplification steps (sequential 2-h incubations at 40°C). Finally, multiple-label probes were hybridized with the specific amplifiers (1 h at 40°C), and samples were run on an LSR Fortessa 4-laser flow cytometer (Becton, Dickinson).

In these experiments, isolated CD4^+^ T cells were stained with 2 different antibody panels (Fig. S3). The first panel comprised the following antibodies for cell surface staining: CD3 (AF700; BioLegend), CCR7 (PE-CF594; BD Biosciences), CD45RO (BV605; BioLegend), CD27 (FITC; BD Biosciences), CD95 (PE-Cy5; BD Biosciences), CD20 (BV786; BD Biosciences), CD32 (PE-Cy7; BioLegend), CD69 (PE; BD Biosciences), HLA-DR (BV711; BD Biosciences). Ki-67 (BV510; BD Biosciences) was used for intracellular staining. The second panel comprised the following antibodies: CD3 (AF700; BioLegend), CD45RO (BV605; BioLegend), CD2 (PE-Cy5; BD Biosciences), PD-1 (PE-Cy7; BD Biosciences), CXCR5 (AF488; BD Biosciences), CD69 (PE; BD Biosciences), HLA-DR (BV711; BD Biosciences), and Ki-67 (BV510; BD Biosciences). The expression of HIV-1 RNA transcripts was analyzed with target-specific AF647-labeled probes, and cell viability was determined using a violet viability dye for flow cytometry (live/dead fixable violet dead cell stain kit; Invitrogen). Negative controls were included in all experiments with cells from non-HIV-1-infected donors. The normalized percentage of HIV-1 RNA expression was calculated for each subpopulation by subtracting the mean value of the negative control from the signal obtained with the analyzed sample.

10.1128/mBio.03078-21.3 FIG S3 A representative example of the gating strategy used for analyses in Fig. 2C. Download FIG S3, TIF file, 0.3 MB. Copyright © 2021 Gálvez et al. 2021 Gálvez et al. https://creativecommons.org/licenses/by/4.0/ This content is distributed under the terms of the Creative Commons Attribution 4.0 International license.

### Intact proviral DNA assay.

Intact provirus in each subpopulation was assessed by intact provirus DNA assay (IPDA) when sample was available. IPDA is based on a duplex ddPCR targeting 2 regions in the viral genome that are present in most intact proviruses, namely, the HIV-1 packaging signal (Ψ) and the Rev responsive element in envelope (*Env*) (21). *RPP30* was targeted to determine the cell number and DNA shearing. The number of intact proviruses was determined based on double positives for Ψ and *Env*.

### Residual viremia by ultrasensitive viral load assay.

Residual viremia (HIV-1 RNA) was measured by ultracentrifugation in 9 ml of plasma at 170,000 × *g* at 4°C for 30 min, followed by viral RNA extraction using the m2000sp Abbot RealTime HIV-1 (34) assay device and laboratory-defined software applications from the instrument. HIV-1 RNA copies in the low range were determined based on an in-house calibration curve set of standards (range, 10 to 10^3^ absolute copies) (35), which had previously been validated using a standard HIV-1 DNA control from the WHO in the range of 0.5 to 128 copies per mL. The limit of detection was calculated relative to the plasma volume used in each sample (down to 0.5 HIV-1 RNA copies/mL when using 9 mL of plasma).

### Cytokine and chemokine plasma levels.

Concentrations of the cytokines and chemokines IFN-γ, IL-12p70, IL-17a/CTLA8, IL-1b, IL-2, IL-7, IP10/CXCL10, MCP1/CCL2, MIP1β/CCL4, and TNF were quantified in 25 μL of plasma using a bead-based multiplex immunoassay (Milliplex; Merck Millipore) according to the manufacturer’s recommendations. Measurements were performed using a Luminex 100 instrument (Luminex Corp.) and analyzed using a standard curve for each cytokine.

### Statistical analysis.

Data are summarized as box and whisker plots indicating the median, interquartile range, and minimum and maximum values. Spearman correlation coefficients were calculated to analyze correlations between study variables. Differences were tested for statistical significance using the Wilcoxon rank sum test or Friedman’s test followed by the Benjamini-Hochberg false-discovery rate adjustment for multiple comparisons. The analyses were performed with R (v3.4) and GraphPad (v8.4).
