# Supplementary figures and images for: Atlas of the HIV-1 Reservoir in Peripheral CD4 T Cells of Individuals on Successful Antiretroviral Therapy
Source: mBio. 2021 Nov 30;12(6):e03078-21. doi: 10.1128/mBio.03078-21 (PMC8630536; doi:10.1128/mBio.03078-21)

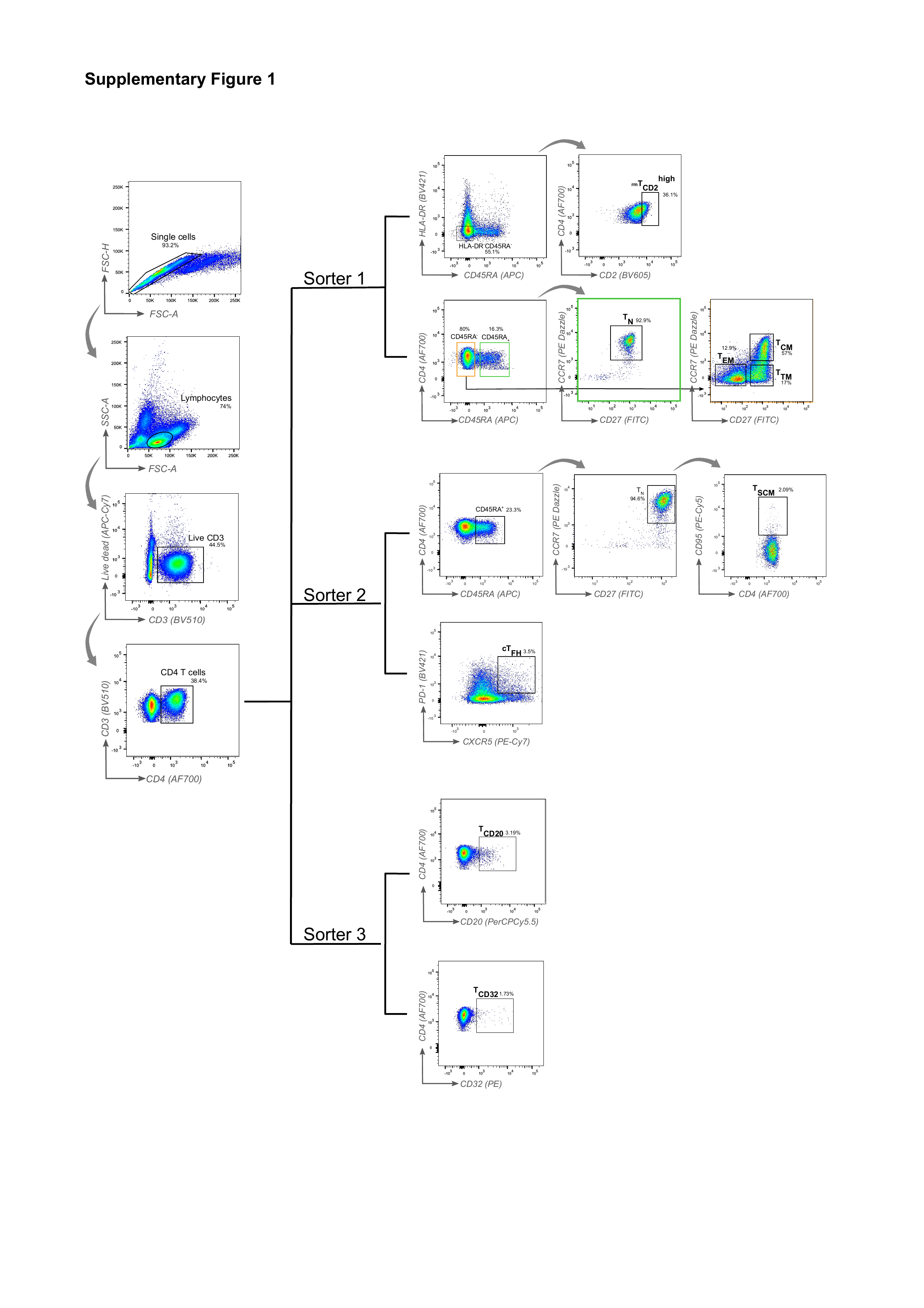

Supplement: FIG S1 [file mbio.03078-21-sf001.tif]

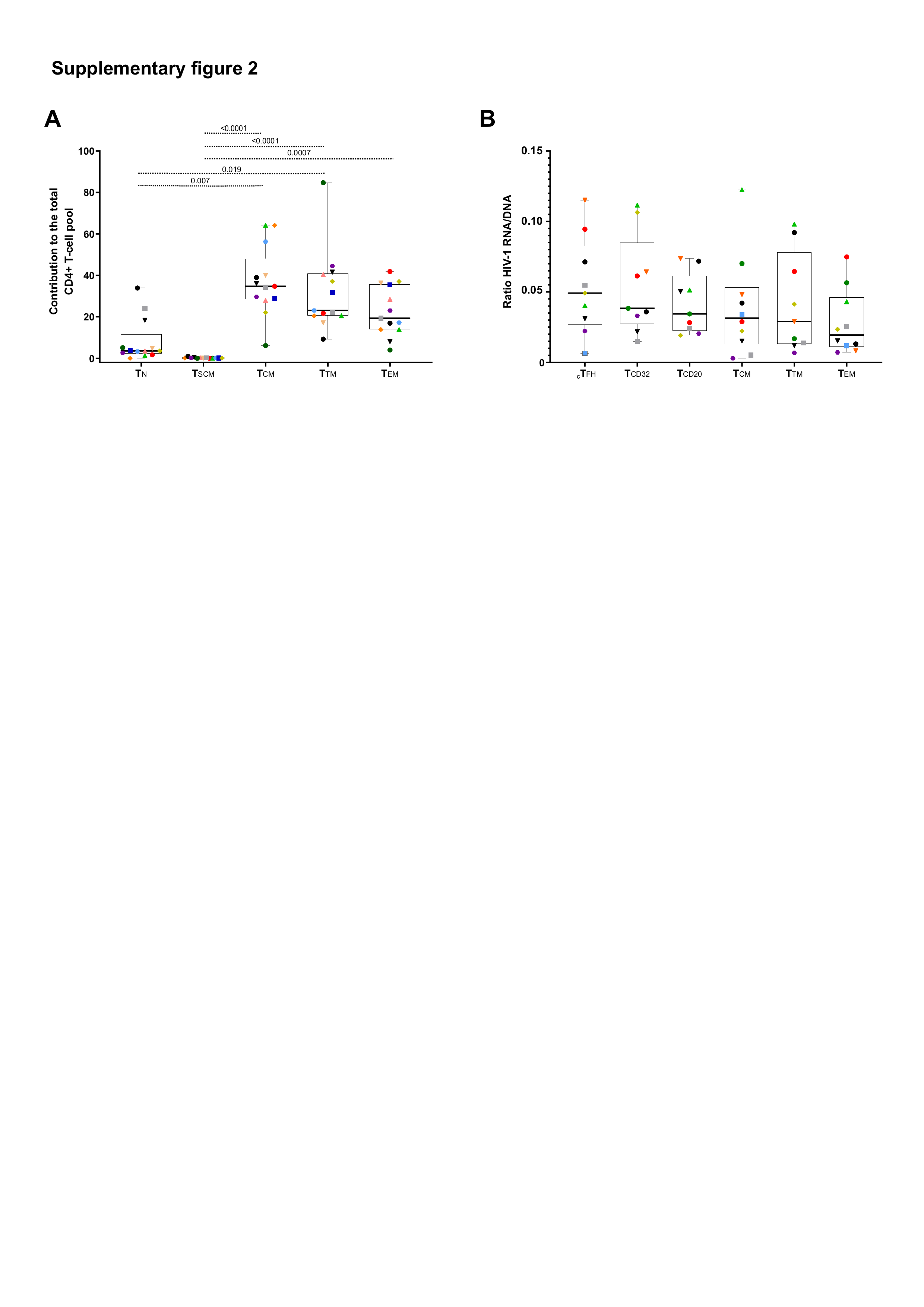

Supplement: FIG S2 [file mbio.03078-21-sf002.tif]

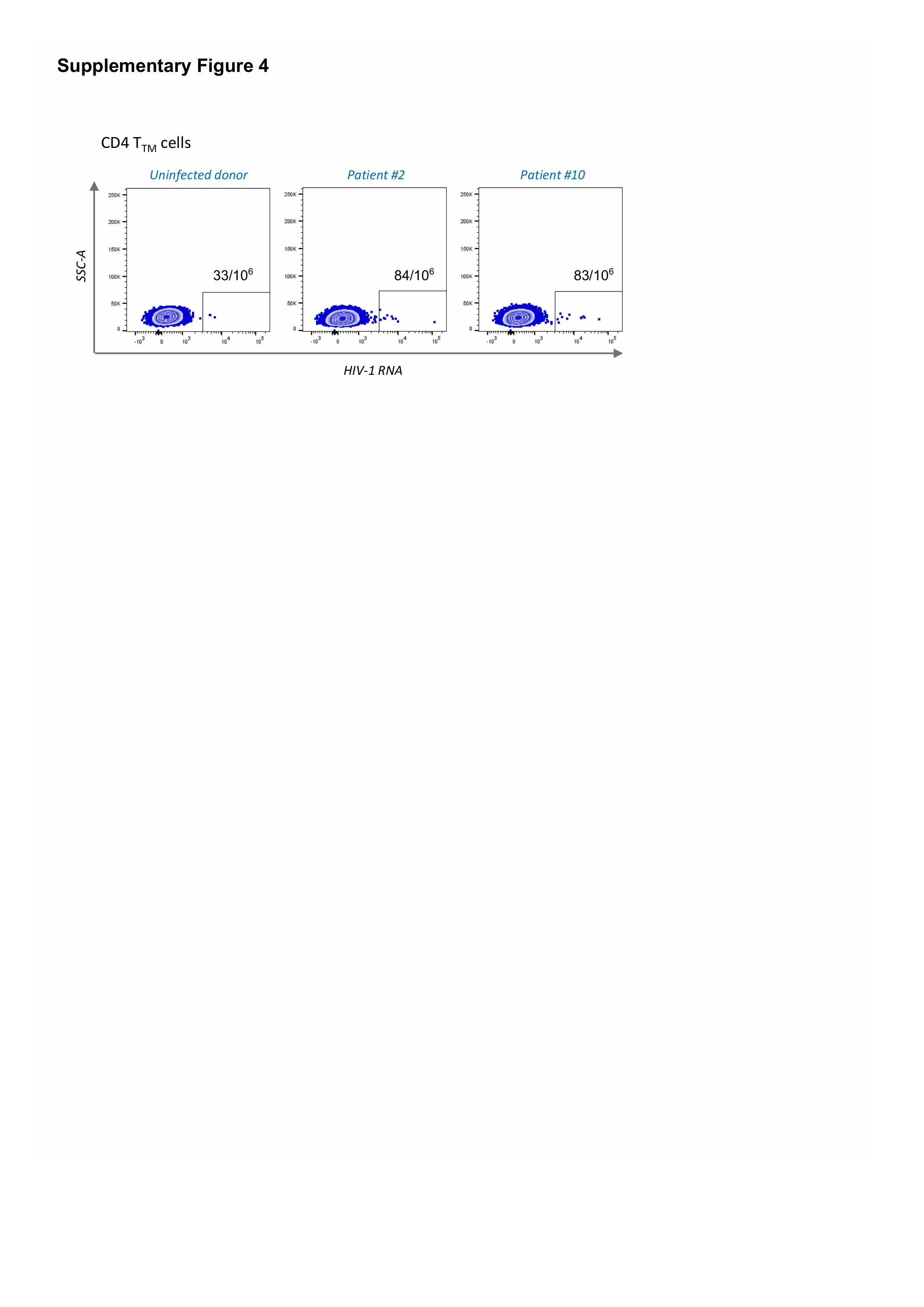

Supplement: FIG S4 [file mbio.03078-21-sf004.tif]

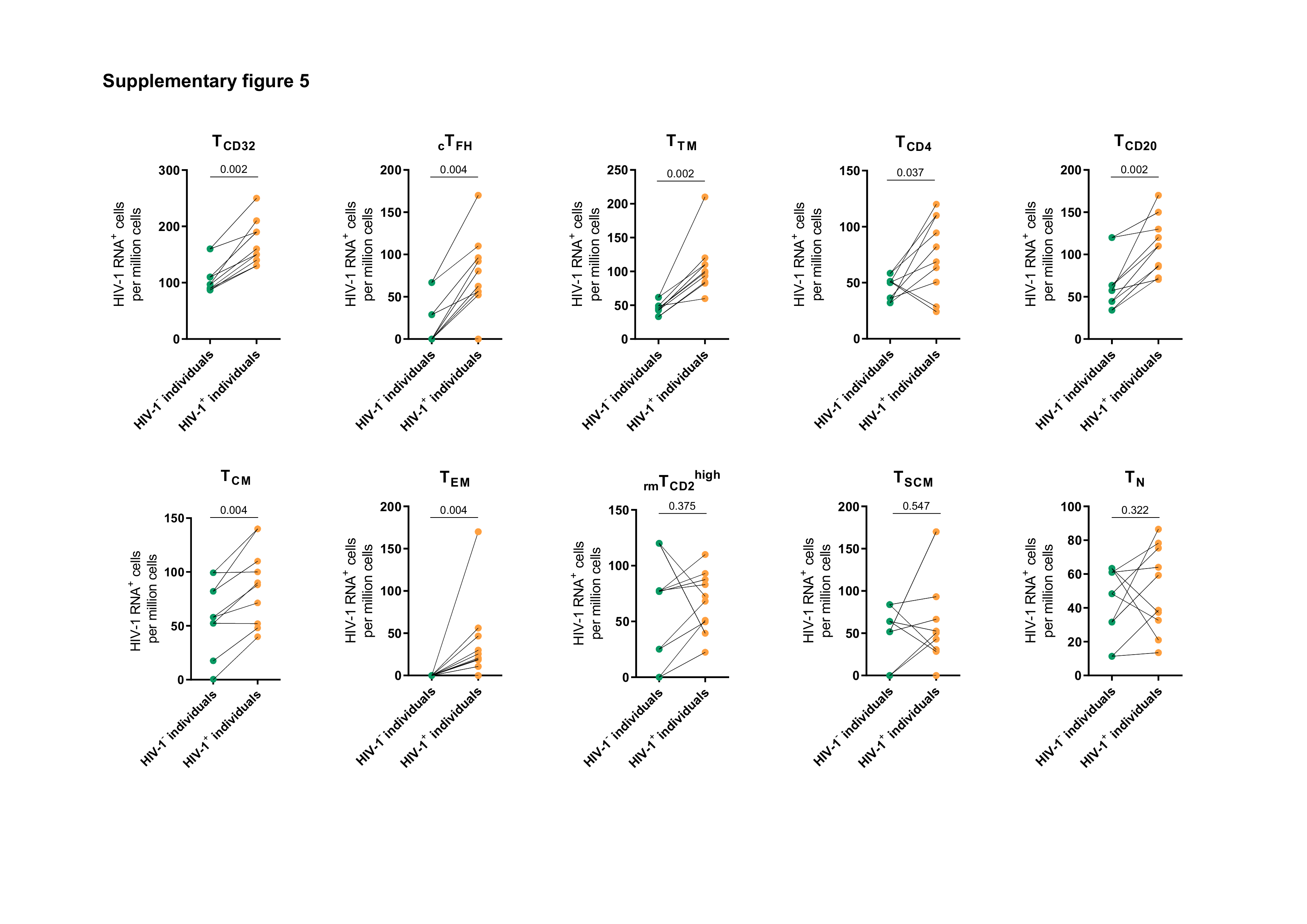

Supplement: FIG S5 [file mbio.03078-21-sf005.tif]

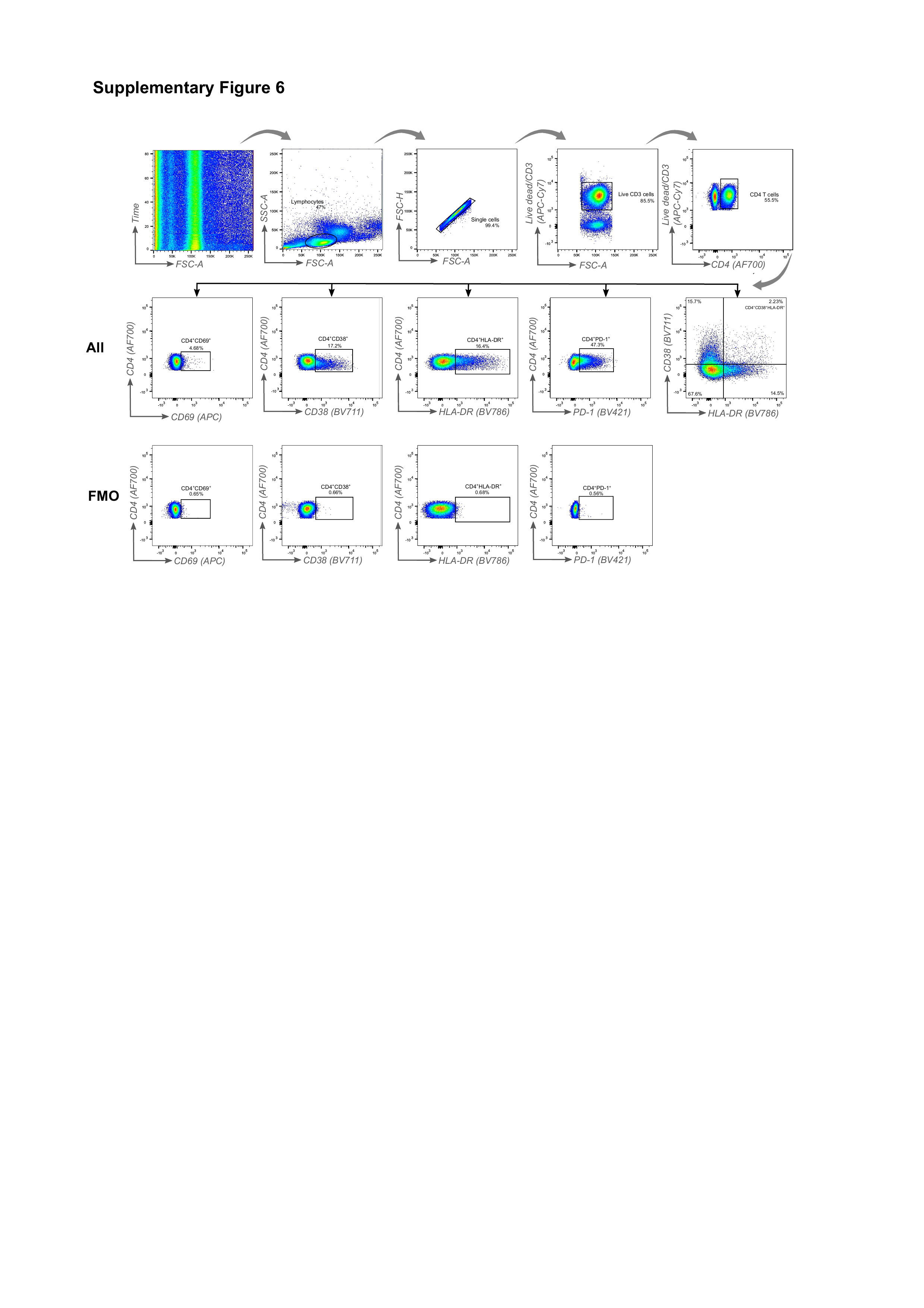

Supplement: FIG S6 [file mbio.03078-21-sf006.tif]

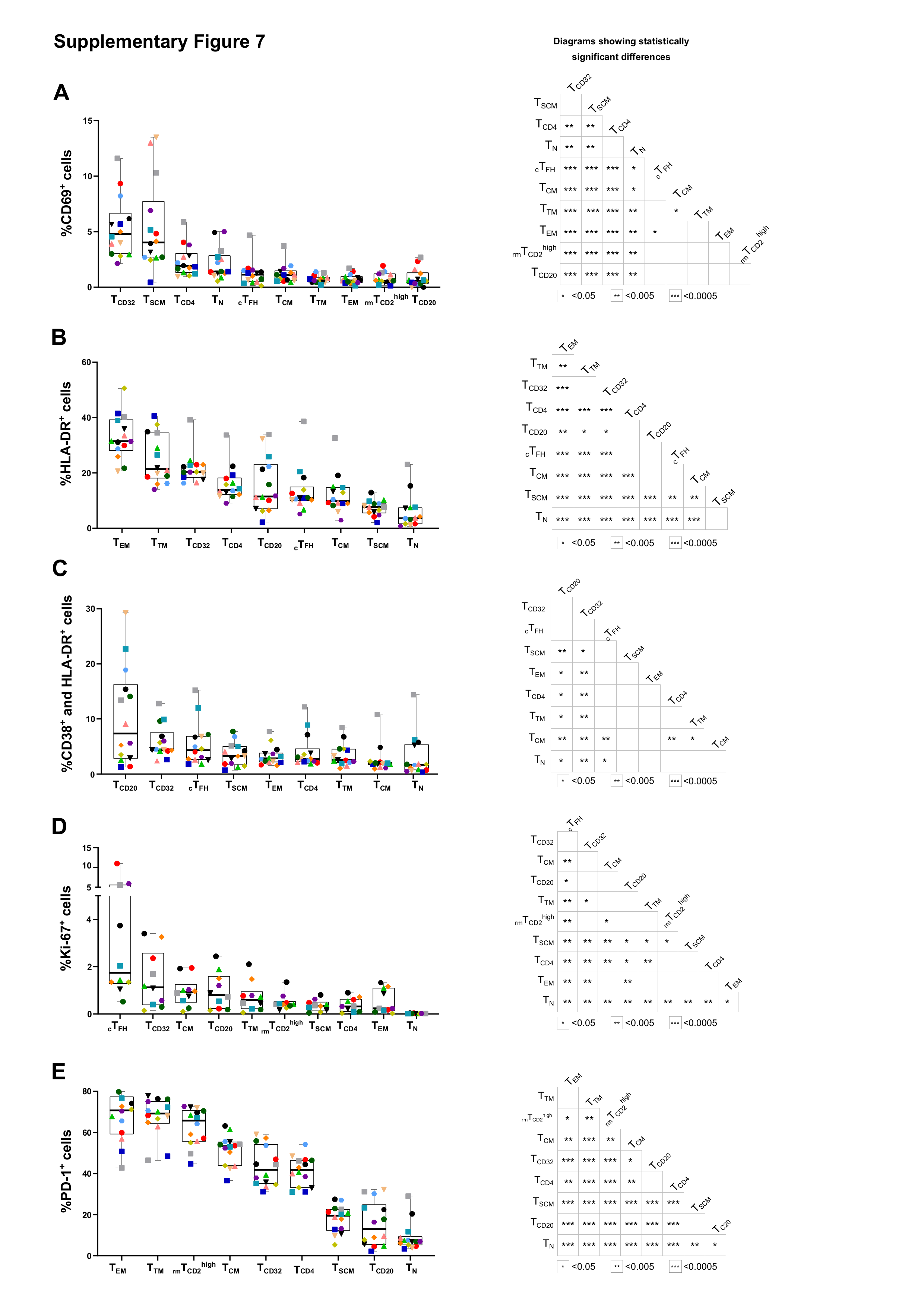

Supplement: FIG S7 [file mbio.03078-21-sf007.tif]

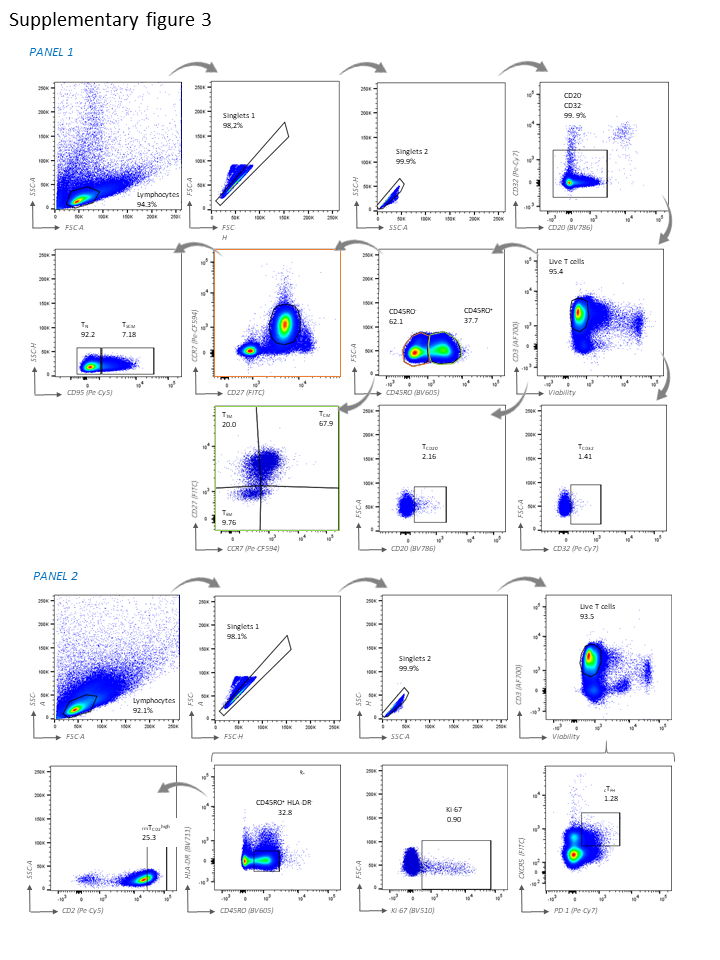

Supplement: FIG S3 [file mbio.03078-21-sf003.tif]
