# Supplementary material for: Atlas of the HIV-1 Reservoir in Peripheral CD4 T Cells of Individuals on Successful Antiretroviral Therapy
Source: mBio. 2021 Nov 30;12(6):e03078-21. doi: 10.1128/mBio.03078-21 (PMC8630536; doi:10.1128/mBio.03078-21)
Supplement: TABLE S1 [file mbio.03078-21-st001.docx]

**Supplementary Table 1. Analysis of the correlation between total HIV-1 DNA, clinical parameters, HIV-1 expression and cytokines/chemokines.**

|  | **T_TM_** | | **T_EM_** | | **_rm_T_CD2_^high^** | | **T_CD20_** | | **_c_T_FH_** | | **T_CM_** | | **T_CD32_** | | **T_CD4_** | | **T_SCM_** | | **T_N_** | |
| --- | --- | --- | --- | --- | --- | --- | --- | --- | --- | --- | --- | --- | --- | --- | --- | --- | --- | --- | --- | --- |
|  | **rho** | **p.val** | **rho** | **p.val** | **rho** | **p.val** | **rho** | **p.val** | **rho** | **p.val** | **rho** | **p.val** | **rho** | **p.val** | **rho** | **p.val** | **rho** | **p.val** | **rho** | **p.val** |
| **Clinical variables** |  |  |  |  |  |  |  |  |  |  |  |  |  |  |  |  |  |  |  |  |
| Time from diagnosis (years) | -0.42 | 0.16 | -0.05 | 0.88 | -0.18 | 0.53 | 0.15 | 0.62 | 0.35 | 0.21 | -0.22 | 0.44 | -0.13 | 0.68 | -0.07 | 0.82 | -0.18 | 0.55 | -0.09 | 0.77 |
| Minimum CD4 cell count | -0.13 | 0.68 | -0.51 | 0.07 | -0.41 | 0.15 | -0.54 | 0.06 | -0.48 | 0.09 | -0.41 | 0.15 | -0.43 | 0.14 | -0.49 | 0.08 | -0.33 | 0.27 | -0.31 | 0.28 |
| Years with undetectable VL | 0.08 | 0.79 | 0.41 | 0.15 | 0.02 | 0.96 | 0.48 | 0.10 | ***0.56*** | ***0.04*** | 0.36 | 0.20 | 0.42 | 0.16 | 0.46 | 0.10 | 0.34 | 0.26 | 0.28 | 0.33 |
| Maximum viral load | 0.15 | 0.62 | 0.34 | 0.24 | 0.02 | 0.96 | 0.21 | 0.49 | 0.35 | 0.23 | 0.30 | 0.30 | ***0.58*** | ***0.04*** | 0.37 | 0.20 | 0.41 | 0.16 | 0.53 | 0.06 |
| **Virological variables** |  |  |  |  |  |  |  |  |  |  |  |  |  |  |  |  |  |  |  |  |
| usVL (copies/ml plasma) | 0.37 | 0.21 | ***0.70*** | ***0.01*** | 0.35 | 0.22 | ***0.57*** | ***0.04*** | ***0.70*** | ***0.01*** | 0.50 | 0.07 | ***0.69*** | ***0.01*** | 0.53 | 0.05 | ***0.71*** | ***0.01*** | ***0.66*** | ***0.01*** |
| %HIV-1 RNA^+^ cells | ***0.70*** | ***0.04*** | 0.41 | 0.25 |  |  | 0.68 | 0.05 | 0.41 | 0.25 | 0.28 | 0.44 | 0.22 | 0.58 | ***-0.66*** | ***0.04*** |  |  |  |  |
| **Cytokines & chemokines** |  |  |  |  |  |  |  |  |  |  |  |  |  |  |  |  |  |  |  |  |
| IFNɣ | -0.25 | 0.40 | -0.46 | 0.10 | -0.18 | 0.53 | -0.42 | 0.16 | ***-0.59*** | ***0.03*** | -0.40 | 0.15 | ***-0.61*** | ***0.03*** | -0.47 | 0.09 | ***-0.57*** | ***0.05*** | -0.43 | 0.12 |
| IL12p70 | 0.19 | 0.53 | -0.06 | 0.84 | 0.22 | 0.44 | -0.01 | 0.97 | -0.16 | 0.59 | *-0.10* | *0.73* | -0.25 | 0.41 | -0.12 | 0.67 | -0.04 | 0.89 | -0.14 | 0.64 |
| IL17a/CTLA8 | ***-0.73*** | ***0.01*** | -0.48 | 0.08 | ***-0.56*** | ***0.04*** | -0.41 | 0.17 | -0.14 | 0.62 | ***-0.53*** | ***0.05*** | -0.54 | 0.06 | -0.43 | 0.13 | ***-0.59*** | ***0.03*** | -0.26 | 0.37 |
| IL1b | 0.09 | 0.76 | -0.12 | 0.67 | 0.28 | 0.33 | -0.17 | 0.58 | -0.17 | 0.57 | -0.12 | 0.69 | -0.29 | 0.34 | -0.16 | 0.58 | -0.10 | 0.75 | -0.29 | 0.31 |
| IL2 | 0.20 | 0.52 | -0.07 | 0.82 | 0.04 | 0.90 | 0.09 | 0.77 | -0.05 | 0.86 | -0.04 | 0.90 | -0.28 | 0.36 | -0.09 | 0.75 | -0.09 | 0.77 | -0.20 | 0.48 |
| IL7 | 0.19 | 0.53 | 0.04 | 0.89 | 0.20 | 0.49 | -0.05 | 0.88 | 0.07 | 0.82 | -0.08 | 0.80 | -0.23 | 0.45 | -0.13 | 0.67 | 0.05 | 0.86 | -0.12 | 0.68 |
| IP10/CXCL10 | 0.04 | 0.91 | -0.06 | 0.84 | -0.02 | 0.94 | -0.21 | 0.48 | -0.29 | 0.32 | -0.28 | 0.33 | -0.41 | 0.17 | -0.16 | 0.57 | -0.29 | 0.34 | 0.02 | 0.96 |
| MCP1/CCL2 | -0.03 | 0.93 | -0.27 | 0.35 | -0.05 | 0.86 | -0.22 | 0.47 | -0.36 | 0.20 | 0.01 | 0.99 | 0.05 | 0.88 | -0.11 | 0.72 | -0.10 | 0.75 | -0.26 | 0.37 |
| MIP1b/CCL4 | -0.07 | 0.82 | -0.09 | 0.77 | 0.15 | 0.61 | -0.24 | 0.43 | -0.03 | 0.92 | -0.29 | 0.32 | -0.25 | 0.41 | -0.15 | 0.62 | -0.13 | 0.67 | -0.20 | 0.48 |
| TNF | -0.33 | 0.27 | -0.40 | 0.16 | -0.07 | 0.82 | -0.50 | 0.08 | -0.31 | 0.27 | ***-0.58*** | ***0.03*** | ***-0.59*** | ***0.04*** | ***-0.55*** | ***0.05*** | -0.37 | 0.21 | -0.43 | 0.12 |
